# Rapid Prototyping of Plastic Lab-on-a-Chip by Femtosecond Laser Micromachining and Removable Insert Microinjection Molding

**DOI:** 10.3390/mi8110328

**Published:** 2017-11-07

**Authors:** Rebeca Martínez Vázquez, Gianluca Trotta, Annalisa Volpe, Giuseppe Bernava, Vito Basile, Melania Paturzo, Pietro Ferraro, Antonio Ancona, Irene Fassi, Roberto Osellame

**Affiliations:** 1 IFN CNR, Institute for Photonics and Nanotechnologies, National Research Council, Piazza Leonardo da Vinci, 32, 20133 Milan, Italy; roberto.osellame@polimi.it; 2 ITIA CNR, Institute of Industrial Technology and Automation, National Research Council, Via P. Lembo 38F, 70124 Bari, Italy; gianluca.trotta@itia.cnr.it (G.T.); vito.basile@itia.cnr.it (V.B.); 3 IFN CNR, Institute for Photonics and Nanotechnologies, National Research Council, Via Amendola 173, 70126 Bari, Italy; annalisa.volpe@ifn.cnr.it (A.V.); antonio.ancona@uniba.it (A.A.); 4 ISASI CNR, Institute of Applied Sciences & Intelligent Systems “E. Caianiello”, Via Torre Bianca—Istituto Marino Pad. 4 Mortelle, 98164 Messina, Italy; massimo.bernava@gmail.com; 5 ISASI CNR, Institute of Applied Sciences & Intelligent Systems “E. Caianiello”, Via Campi Flegrei 34, 80078 Pozzuoli, Italy; melania.paturzo@cnr.it (M.P.); pietro.ferraro@cnr.it (P.F.); 6 ITIA CNR, Institute of Industrial Technology and Automation, National Research Council, Via A. Corti 12, 20133 Milan, Italy; irene.fassi@itia.cnr.it

**Keywords:** lab-on-a-chip, micro-injection-molding, femtosecond laser micromachining, fluorescent cytometry, cell phone

## Abstract

We have introduced a new hybrid fabrication method for lab-on-a-chip devices through the combination of femtosecond laser micromachining and removable insert micro-injection molding. This method is particularly suited for the fast prototyping of new devices, while maintaining a competitive low cost. To demonstrate the effectiveness of our approach, we designed, fabricated, and tested a completely integrated flow cytometer coupled to a portable media device. The system operation was tested with fluorescent plastic micro-bead solutions ranging from 100 beads/μL to 500 beads/μL. We demonstrated that this hybrid lab-on-a-chip fabrication technology is suitable for producing low-cost and portable biological microsystems and for effectively bridging the gap between new device concepts and their mass production.

## 1. Introduction

The concept of a lab-on-a-chip (LOC) is founded on the integration of several laboratory functions on a single device with dimensions on the scale of millimeters to few centimeters [1,2]. The main goal is to perform chemical or biological synthesis and analysis using very small sample volumes with high reproducibility. To succeed, it is necessary to combine many different technological areas, with microfluidics binding all them together through the control and manipulation of fluids inside small channels [3]. 

An application area where LOCs are attracting great interest and applicability is the detection and counting of single particles and cells, in devices following the principle of flow cytometers [4,5,6]. Flow cytometers are fundamental instruments in biomedical sciences as they are used in routine analysis, like blood cell count, or in more complex assays in cell biology, immunology, oncology, etc. Current commercial flow cytometers have reached table-top size with very high sensitivity and throughput and are capable of performing very complex analyses; however, they have a high cost and very limited portability [7]. Translating this technology to cost-effective and portable hardware could open new perspectives in biomedicine, enabling sophisticated analyses in remote or resource-limited environments. In this framework, microfluidic flow cytometers constitute the best solution. However, to actually have a portable system, not only the microfluidic chip, but also all the instrumentation that makes its operation possible have to be small. The most widespread, powerful, and compact instrumentation available nowadays is represented by mobile telephones, which have now reached an incredible level of performance and which already include many top-level sensors. An effective strategy to develop highly portable systems is thus to combine lab-on-a-chip devices with mobile phones [8,9]. This approach allows one to significantly contain the cost of the system and greatly expand its application; however, it requires smart engineering of the devices to take full advantage of the resources already available in portable electronic devices, such as mobile phones.

Polymer is often chosen as the material to manufacture lab-on-a-chip devices [10] owing to its low cost, easy processing, and wide variety of compositions [11]. In particular, thermoplastic poly(methyl methacrylate) (PMMA) is commonly used when optical sensing is needed, due to its very good transparency in the visible and infrared regions. Moreover, it is quite robust and presents a higher chemical resistance than other polymers [12]. 

Mass replication techniques are natural candidates for the microfabrication of plastic devices and, in particular, microinjection molding (µIM) is quite promising due to its high throughput [13,14]. Unfortunately, it requires the fabrication of expensive masks and molds, which constitutes the weak point of the technology for device prototyping.

Femtosecond laser micromachining (FLM) is a versatile tool with multiple different applications in materials processing [15] and in particular for the fabrication of lab-on-a-chip devices [16,17,18]. Femtosecond laser micromilling is an ablation procedure that causes the vaporization of material in a layer-by-layer fashion because of the interaction between the laser beam and the workpiece being machined. It is gradually emerging as an important technology for applications in rapid prototyping and manufacturing of miniaturized metallic, semiconductor, glass, and ceramic components or devices. Femtosecond laser pulses provide high precision and reduced thermal damages and/or structural modifications compared to longer laser pulses [19]. Moreover, femtosecond lasers can also be used for welding different transparent materials at their interface, exploiting localized melting and re-solidification below the ablation threshold [20].

In this work, we propose a novel hybrid approach that combines FLM and µIM in order to overcome the weak aspects of each technology separately. Moreover, to manufacture the plastic microfluidic chip, we use a smart variation of microinjection molding that introduces the concept of removable and tailored inserts, which further reduces the cost of the mold fabrication and increases the flexibility of this technology. FLM is initially involved in the manufacturing of micro-sized features in the metal inserts by laser ablation and finally in the bonding of the two plastic slabs that constitutes the microfluidic device, thus avoiding the use of glue or other interface materials that could contaminate the measurements.

To demonstrate the potential of this technology, we introduce a cost-effective and highly portable PMMA prototype that is interfaced with a mobile phone to implement an optofluidic cytometry platform. This system performs fluorescence detection and counting of particles by exploiting the light emitting diode (LED), already integrated in the mobile phone, for the fluorescence excitation, as well as the integrated camera (CMOS) for its imaging. Additional optical components and the microfluidic chip are held by a specific 3D-printed plastic support that fits onto the mobile phone. A dedicated application software (app) has been developed for image processing and particle counting by automatic particle recognition on the acquired movies. All the data processing is performed on the portable device, allowing its operation in the absence of a connection to a computer or to the internet.

## 2. Materials and Methods 

**Figure 1:**
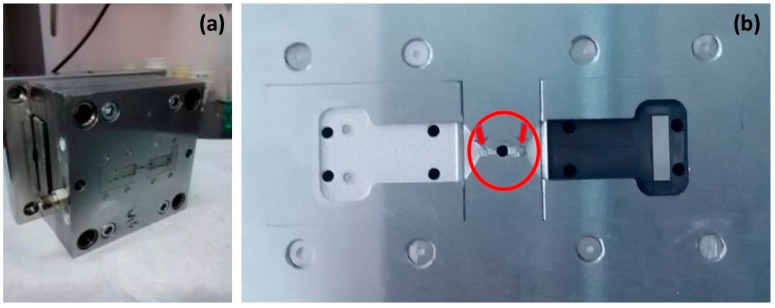
(**a**) Picture of the mold; (**b**) Overview of the mold inserts for the production of the plates, where the interchangeable pins are highlighted.

The removable insert mold has to be carefully designed in order to fulfil the characteristic of a standard µIM machine on the one hand, and to produce the features of the final microfluidic chip on the other. It consists of two cavities that will allocate the interchangeable steel inserts (steel grade 28NiCrV5, DIN 1.2737) that constitute the negative of the final PMMA slabs (see Figure 1a). This design increases the versatility of the approach; each micro-feature can be easily replaced independently from the overall mold by simply replacing the related insert. Furthermore, we followed the strategy of making a single sprue with a double symmetric gate facing the inserts in the cavities (Figure 1b). In this way, the cavities are independent from the feeding system and are therefore easily replaceable. Since the volume of polymer required for each cavity (about 125 mm^3^) is close to the limit of the µIM machine capacity (150 mm^3^), a couple of interchangeable pins with different heights are used to alternatively close one of the runners (Figure 1b—circle) so that one cavity is filled at a time. 

Once the overall geometry is selected, a complete numerical simulation of 3D filling and packing of the µIM process is performed using Moldflow Plastics Insight^®^. The entire part was modeled, including sprue and runners.

**Figure 2:**
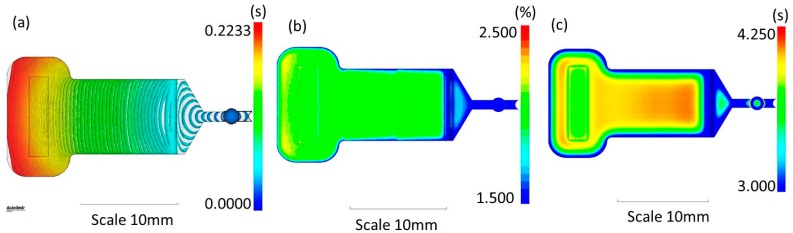
Numerical simulation of 3D filling and packing of the molten polymer in the mold during the µIM process. (**a**) Polymer filling distribution (time contour plot) (s); (**b**) Volumetric shrinkage in the range 1.5–2.5%; (**c**) Time to reach ejection temperature in the range 3–4.25 s.

In Figure 2, we show the results for the parameters that were optimized. These parameters include the polymer filling distribution, through the analysis of the contour plot (Figure 2a); the volumetric shrinkage, ensuring that the shrinkage distribution remains in the expected range (between 2% and 3%, Figure 2b); and the time to reach the ejection temperature, observing whether the component reaches the ejection time as uniformly as possible (Figure 2c).

The analysis of the process parameters evidences a uniform distribution of the flow through the cavity (fountain flow) with good distance of the contour lines, a necessary condition to completely fill the cavity and to guarantee a good effect of the packing phase. The distribution of the shrinkage along the whole component is in the range expected for the PMMA, and this guarantees a stable final component with reduced warpage effects and that is easy to be demolded. 

The manufacturing of the steel inserts is made by a combination of FLM and traditional surface machining techniques. First, the features that are common to all possible microfluidic layouts, such as the ejection systems and the microfluidic access holes, are fabricated by traditional milling and micro electro discharge machining (µEDM). Second, the microfluidic channel, whose layout is specific to each device and that contains the finest features, is fabricated by FLM. The latter is in fact a faster and more versatile approach for machining 3D microstructures on metal inserts, with minimal thermal and mechanical damage of the workpiece. 

For FLM, we use an ultrafast fiber laser amplifier from Active Fiber Systems GmbH (Jena, Germany) based on the chirped pulse amplification technique (CPA). The laser source delivers an almost diffraction-limited beam (M2 ~ 1.25) at a wavelength of 1030 nm with a pulse duration in the range of 650 fs to 20 ps, a repetition rate varying from 50 kHz to 20 MHz, a maximum pulse energy of 100 μJ, or maximum average power of 50 W. The linearly polarized beam exiting from the laser source is firstly converted into circularly polarized light by a quarter-wave-plate and then focused and moved onto the target surface through a galvo-scan head (model Hurryscan from SCANLAB GmbH, Puchheim, Germany) equipped with a F-theta lens of 100 mm focal length. The resulting beam spot size on the metal surface is about 25 µm. The laser irradiation is performed in a line-by-line scanning, at a laser repetition rate of 50 kHz, with a power of 400 mW and 7.05 TW/cm^2^ peak intensity, at the focus of the F-theta lens moving the beam at a speed of 600 mm/s with a separation between adjacent scanned lines of 2 µm. These parameters were carefully optimized in order to achieve a surface roughness on ablated steel of 590 nm (rms) and the highest fidelity to the design of the microfluidic network [21].

After the production of the two PMMA slabs by µIM, they are welded with the same femtosecond laser. The two samples are clamped in order to achieve an air gap of a few micrometers range in the welding area, namely when interference effects can be observed. The laser source is operated at 5 MHz with a pulse energy of 0.4 μJ and 1.57 TW/cm^2^ peak intensity. The beam is focused through a 0.3 numerical aperture lens at the interface between the two plates. The translation speed is set at 0.1 mm/s. The welding joint consisted of a contour around the microfluidic channel, which is obtained by moving the laser beam along a square spiral path following the desired trajectory [22]. 

**Figure 3:**
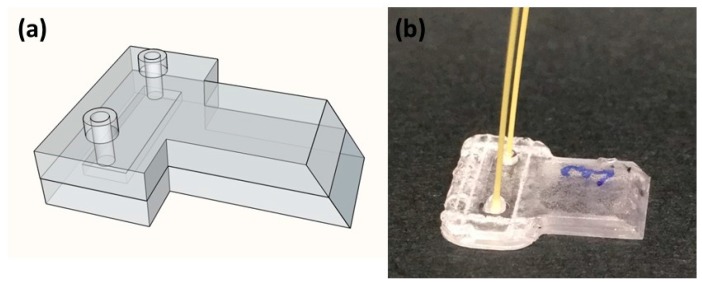
(**a**) 3D scheme of the microfluidic chip; (**b**) picture of the final chip, bonded by femtosecond laser irradiation, and with the peek tubes plugged inside the access holes.

The scheme of the chip for the integrated flow cytometer is shown in Figure 3a. The overall geometry of the PMMA slabs follows a T shape; the top branch contains the microfluidic channel and the access holes, while the long branch has a 45° edge and is used as a waveguide to deliver light from the phone LED to the channel. The dimensions of the microfluidic channel are 6.75 mm in length, 2 mm in width, and 50 µm in height. The low microchannel height will force large particles to flow at the same depth, thus allowing all of them to be in focus when imaging that plane. On the other hand, the large width will provide a reasonable flow rate to keep measurements short (a few minutes). A picture of the assembled microfluidic chip is shown in Figure 3b; the trace of the laser bonding (a closed rectangle) and the plugged peek tubes (Outer diameter 360 µm, Upchurch Scientific^®^ PEEK™, IDEX Health & Science LLC, Oak Harbor, WA, USA) that will be used to deliver the liquid sample to the microfluidic channel are clearly visible.

To perform the fluorescence cytometer measurements, we use an iPod (6th generation, Apple Inc., Cupertino, CA, USA) as the portable media device as it has the same dimensions and sensors of a mobile phone, apart from the telephone connection, which is not necessary in our experiments. It has a double LED and a 5 Mpixel color RGB CMOS sensor that we will use respectively to excite the fluorescence and capture the movies of the flowing specimens. The device has a built-in lens (with a focal length of f_1_ = 3 mm) in front of the CMOS camera that we will also exploit in the imaging of the flowing sample. The microfluidic chip and the additional optical components of our cytometry platform are held together and connected to the mobile device by means of a 3D plastic interface fabricated with a 3D printer (Formlabs 1 + 3D).

The performance of the final cytometry platform is tested using different aqueous solutions of fluorescent polystyrene beads (20 µm diameter, Phosphorex Inc, Hopkinton, MA, USA, targeted with the fluorescence dye Orange). The size of these beads is comparable to that of tumor cells widely studied in the literature [23]. We prepared suspensions in deionized water to obtain bead solutions ranging from 100 beads/μL to 500 beads/μL. These solutions are inserted in the microfluidic channel by means of a standard syringe, actuated by hand, with a typical flow ratio of 6 μL/min.

## 3. Integrated Flow Cytometer

**Figure 4:**
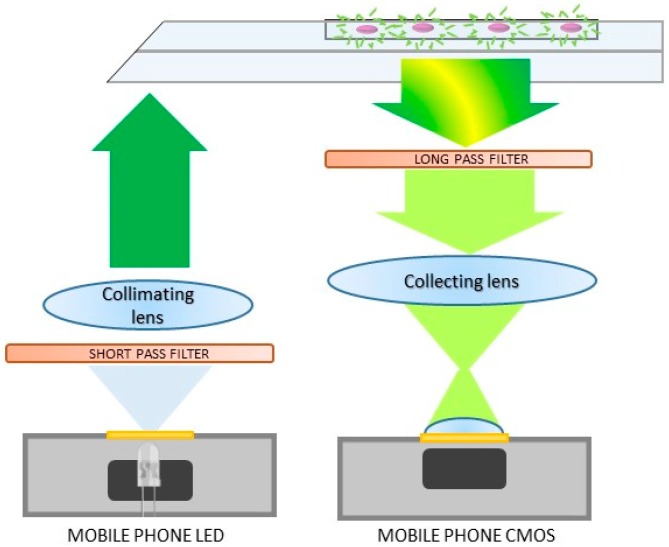
Optical scheme employed in the fluorescent cytometer to excite and collect the fluorescence signal by exploiting the built-in LED and CMOS camera of a mobile device.

The compact fluorescent cytometry unit follows the schematic layout shown in Figure 4. The white light from the LED is filtered by a short pass filter (Thorlabs FESH0550) and collimated by a 3.3-mm focal length aspheric plastic lens (Thorlabs CAY033, numerical aperture 0.4). It is then reflected on the 45° edge of the microfluidic chip by total internal reflection and redirected to the horizontal direction. Due to the refractive index contrast between the PMMA slabs (1.49) and air (1), the LED light is confined inside the PMMA slabs due to total internal reflection and it is delivered to the imaging volume inside the microfluidic channel. The fluorescence emitted by the specimens is then filtered to remove the remaining excitation light (long pass filter, Thorlabs FGL 550). 

**Figure 5:**
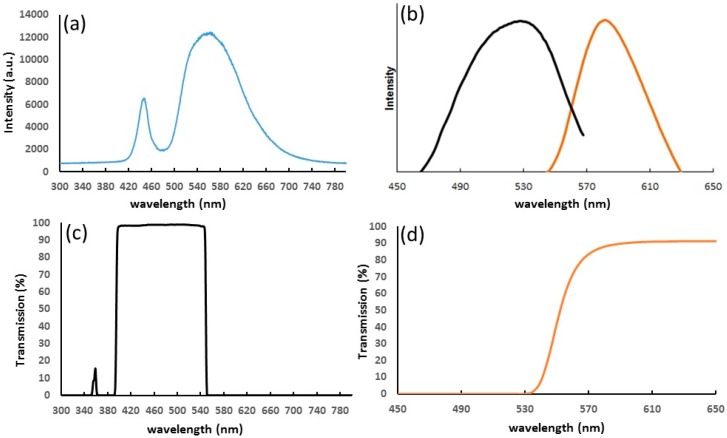
(**a**) iPod LED emission spectra; (**b**) absorption (black) and emission (orange) spectra of orange phosphorex microspheres (from [24]); transmission spectra of filters: (**c**) short pass Thorlabs FESH0550 and (**d**) long pass Thorlabs FGL 550 (from [25]).

The optical filters configuration is designed by taking into account the optical characteristic of the integrated LED and the fluorescent specimen under study. Figure 5a reports the emission spectra of the LED recorded with a high-resolution spectrometer (Ocean Optics, HR2000+, Largo, FL, USA). It consists of two bands covering almost the complete visible spectra, thus in principle our platform is able to perform measurements at different exciting wavelengths just by changing the short pass filter. In Figure 5b, we report the excitation and emission spectra of the fluorescent micro-beads employed to test the system. Their absorption band is centered at around 530 nm wavelength and their emission is at around 580 nm. The short pass and long pass filters (see transmission spectra in Figure 5c,d) are consequently chosen in order to maximize the fluorescence excitation and collection of micro-bead solutions.

The design of the imaging system exploits the built-in lens in front of the CMOS sensor (which has a focal length of f_1_ ~ 3 mm) and a second aspheric plastic lens, with a focal length of f_2_ ~ 4.6 mm (CAY046, numerical aperture 0.4) placed under the microfluidic channel. This combination gives a magnification of 1.5 (f_1_/f_2_) between the sample plane (located at a distance f_2_ with respect to the collecting lens) and the CMOS sensor, yielding an effective pixel size of 2.3 µm. 

**Figure 6:**
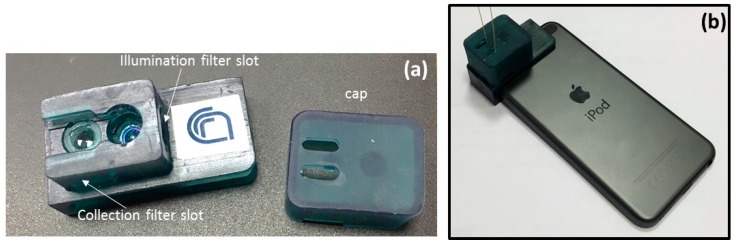
(**a**) Picture of the plastic 3D-printed holder and (**b**) view of the whole system coupled to the iPod.

In the final assembly, the lenses are fixed at the required distances from the chip and the LED and CMOS camera by a 3D-printed plastic holder (see Figure 6a). The filters are also located in specific slots, allowing their easy change in order to use the device for the detection of several different fluorescent specimens. The plastic lab-on-chip is also removable and easily substituted in the holder. A final cap (with small holes for the peek tubes) allows covering the whole system in order to reduce background noise from ambient light. A picture of the entire fluorescent cytometer, coupled to the portable device, is shown in Figure 6b.

We developed a specific application software (app) that acquires movies of the fluorescent flowing particles, performs digital image processing to recognize cell or particles, automatically counts them, and calculates their density for a given sample. All the processing is implemented on the portable device, allowing its operation even in absence of an internet connection or of additional processing units, which were necessary in previous demonstrations of flow cytometry on a cell phone [26]. If needed, the movies can also be transferred to a computer for further digital analysis.

**Figure 7:**
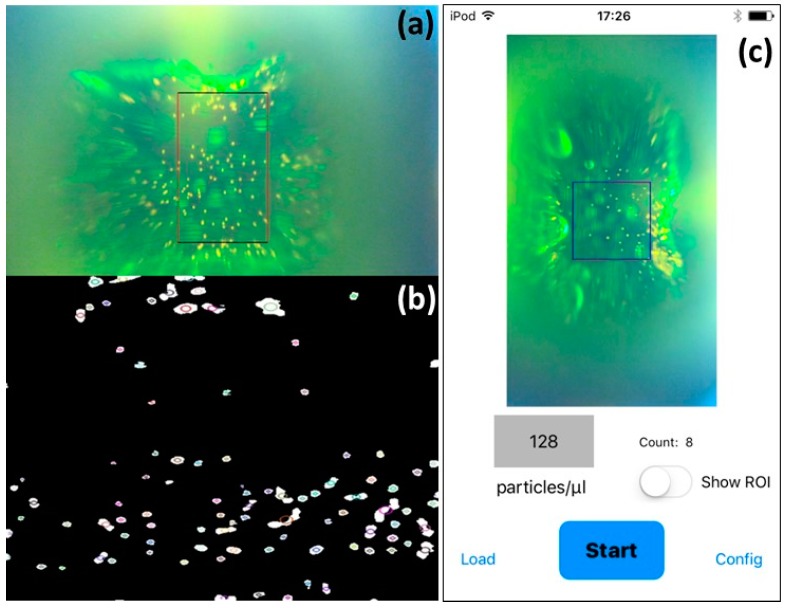
(**a**) Single frame of one video captured with the flow cytometry platform and (**b**) selected ROI with the identification of the flowing particles (circles around white spots); (**c**) Software application screenshot; the particle concentration is given at the end of the measurement.

A video of the fluorescence emission from the flowing sample is continuously recorded. Due to optical aberrations, only the central region of the image is focused and useful for counting purposes, so we define a Region of Interest (ROI), whose volume is known, where the software will detect and count particles. When the user pushes the start button, the software starts to detect and count particles inside the user-defined ROI. To do so, the software converts the image from Red, Green, and Blue (RGB) to Hue Saturation Value (HSV) mode and a digital filter is used between a minimum and a maximum hue value to further remove the green background (see Figure 7a,b). Only the ROI is considered in the resulting image and blobs are extracted and counted according to the following algorithm:

1. Convert the source image to binary images by applying thresholding.
2. Extract connected components from every binary image by finding contours and calculating their centers.
3. Group centers from several binary images by their coordinates.
4. From the groups, estimate final centers of blobs and their radii and return as locations and sizes of keypoints.
5. Remove the blobs that are too small.

In order to evaluate the cases of particles close to each other so as to form a single big blob, the algorithm calculates the average size of the blobs, and all the larger size blobs are counted proportionally.

The app continuously gives a counting value. Figure 7c reports the screenshot of the iPod after pushing OFF, when it stops the counting and immediately calculates the concentration in particles/microliter. If compared with the tracking particle method, our procedure might give a higher counting error as it does not take into account accidental events like particles that change their trajectory or stop during the measurement (and thus will count them more than once). To overcome this problem, it is mandatory to have a microfluidic chip with a perfect laminar flow, as in our case. In this situation, our approach is stronger as it allows performing measurements without an auxiliary computer, increasing “point of care” capabilities in comparison to devices already present in the literature [8,9]. 

## 4. Conclusions 

In this work, we have demonstrated that the combination of microinjection molding with interchangeable inserts and femtosecond laser micromachining constitutes a flexible and cost-effective technique for prototyping lab-on-a-chip devices. We have designed, fabricated, and tested a lab-on-a-chip for counting fluorescent micrometric particles. We have developed a complete platform that, coupled with a mobile phone, allows performing point of care fluorescent analysis. We expect that in the future, this platform will be tested with real biological samples in order to be used in application fields where flow cytometers are already employed. 

We are confident that this device will pave the way for a new generation of compact and disposable lab-on-a-chip devices with point of care applications.
